# Simulating drug penetration during hyperthermic intraperitoneal chemotherapy

**DOI:** 10.1080/10717544.2020.1862364

**Published:** 2021-01-11

**Authors:** Daan R. Löke, Roxan F. C. P. A. Helderman, Nicolaas A. P. Franken, Arlene L. Oei, Pieter J. Tanis, Johannes Crezee, H. Petra Kok

**Affiliations:** a Department of Radiation Oncology, Cancer Center Amsterdam, Amsterdam UMC, University of Amsterdam, Amsterdam, The Netherlands; b Laboratory for Experimental Oncology and Radiobiology, Center for Experimental and Molecular Medicine, Cancer Center Amsterdam, University of Amsterdam, Amsterdam, The Netherlands; c Department for Surgery, Amsterdam UMC, University of Amsterdam, Cancer Center Amsterdam, Amsterdam, The Netherlands

**Keywords:** Hyperthermic intrapertioneal chemotherapy (HIPEC), computational fluid dynamics (CFD), computational modeling, cancer biology, drug dynamics, interstitial fluidpressure (IFP), treatment planning software

## Abstract

Hyperthermic intraperitoneal chemotherapy (HIPEC) is administered to treat residual microscopic disease after debulking cytoreductive surgery. During HIPEC, a limited number of catheters are used to administer and drain fluid containing chemotherapy (41–43 °C), yielding heterogeneities in the peritoneum. Large heterogeneities may lead to undertreated areas, increasing the risk of recurrences. Aiming at intra-abdominal homogeneity is therefore essential to fully exploit the potential of HIPEC. More insight is needed into the extent of the heterogeneities during treatments and assess their effects on the efficacy of HIPEC. To that end we developed a computational model containing embedded tumor nodules in an environment mimicking peritoneal conditions. Tumor- and treatment-specific parameters affecting drug delivery like tumor size, tumor shape, velocity, temperature and dose were assessed using three-dimensional computational fluid dynamics (CFD) to demonstrate their effect on the drug distribution and accumulation in nodules. Clonogenic assays performed on RKO colorectal cell lines yielded the temperature-dependent IC50 values of cisplatin (19.5–6.8 micromolar for 37–43 °C), used to compare drug distributions in our computational models. Our models underlined that large nodules are more difficult to treat and that temperature and velocity are the most important factors to control the drug delivery. Moderate flow velocities, between 0.01 and 1** ***m*/*s*, are optimal for the delivery of cisplatin. Furthermore, higher temperatures and higher doses increased the effective penetration depth with 69% and 54%, respectively. We plan to extend the software developed for this study toward patient-specific treatment planning software, capable of mapping and assist in reducing heterogeneous flow patterns.

## Introduction

Patients diagnosed with gastrointestinal or gynecological malignancies in combination with peritoneal metastasis (PM) face a grim prognosis. PM can result from several gastrointestinal or gynecological origins such as colorectal cancer, ovarian cancer and gastric cancer. Patients with PM from colorectal or gastric origin are observed to have an average overall survival of 5–24** **months and 4–8** **months, respectively (Pelz et al., 2010; Thomassen et al., 2014; Huang et al., 2015; Tan et al., 2017). For ovarian cancer patients, the metastatic involvement of the peritoneum reduces the 5-year survival rate from over 90% to just 25–29% (Testa et al., 2018). A debulking cytoreductive surgery, removing the macroscopic tumors, is often the only treatment option for these patients. However, microscopic tumor nodules, individual and/or small isolated groups of cancer cells can still remain present in the peritoneum, risking recurrences after treatment. Hyperthermic intraperitoneal chemotherapy (HIPEC), performed directly after surgery, aims to eradicate this residual microscopic disease.

During HIPEC, chemotherapeutics are dissolved in a carrier solution, most frequently a saline or dextrose solution (Helderman et al., 2019). After heating the fluid to a predetermined temperature level between 39 °C and 43 °C, the perfusate is circulated through an open or closed abdomen for a duration varying between 30** **minutes and 2** **hours. In general, the efficacy of HIPEC treatments depends on eight parameters (Helderman et al., 2019): the type of chemotherapy, chemotherapy concentration, carrier solution, volume of the perfusate, temperature of the perfusate, duration of the treatment, the technique of delivery and patient selection. The choice of chemotherapy depends predominantly on tumor characteristics. Frequently used chemotherapeutics during HIPEC are cisplatin, mitomycin-C and oxaliplatin (Helderman et al., 2019). One of the strong prognostic factors for clinical outcome is the extent of removal of the macroscopic tumor load before HIPEC is administered. Patients undergoing a successful or complete cytoreductive surgery have a longer overall survival than patients receiving an incomplete cytoreductive surgery (Cavaliere et al., 2011). A complete cytoreductive surgery, labeled CCR-0, is defined as the removal of all macroscopic tumor nodules. If visible tumor nodules still remain present in the peritoneum, the cytoreductive surgery is dubbed incomplete, labeled either CCR-1 or CCR-2 with remaining tumor nodules of up to 2.5** **mm or larger than 2.5** **mm, respectively. One of the therapeutic barriers for HIPEC treatments is the limited penetration of chemotherapy into the tumor nodules. The approximate penetration is generally assumed to be only a few millimeters (Kusamura et al., 2008).

The physical processes of both convection and diffusion play an important role in the delivery of drugs during HIPEC. Convective forces are solely responsible for the delivery of the drugs to the tumor surface. Diffusive forces take over when the drugs penetrate the tissues. An important factor that could limit drug penetration in tumor tissue is an increased interstitial fluid pressure (IFP), a known therapeutic barrier in drug delivery in solid tumors (Heldin et al., 2004; Lunt et al., 2008). In healthy tissues, water and larger molecules travel through the interstitium to maintain homeostasis. The convective flow in the interstitium is pressure driven by a pressure gradient over the capillary wall causing an outward flux to drive the convection inside the interstitium toward a drain, for example the lymphatic system. In cancerous tissues, a fully functioning lymphatic system is absent, while a vascular system is still present, albeit in irregular form. Together with other causes such as fibrosis and an irregular vascular system this tends to cause an accumulation of fluid inside the tumor (Heldin et al., 2004). The fluid near the edge of the tumor can flow away, while the fluid near the center of the tumor accumulates causing an increased IFP. The increased IFP results in an outward convective flow, complicating inward drug delivery. Intravenously delivered chemotherapy enters the tumor through the capillaries and diffuse inside the tumor while intraperitoneal delivered chemotherapy is administered from the outside of the tumor, having to diffuse from the exterior to the interior of the tumor. Ultimately the resulting penetration is determined from the balance between the outward convective flow and the inward diffusive force.

Numerical simulations can provide unique insights in various biological processes providing a firm base for the optimization of both radiation- and chemotherapy-based treatments involving hyperthermia. Treatment planning software for radio-frequency-based hyperthermia has made its way into the clinic providing online guidance to clinicians (Kok et al., 2017). Treatment planning software for treatments involving large fluid columns, like HIPEC, incorporating the field of computational fluid dynamics (CFD), is still in its infancy. In the last decade, several studies showed the potential of CFD-approaches in cancer treatments. Bhandari et al. (2017, 2018) developed a CFD-based drug transport model, incorporating realistic vasculature structures and performed several studies predicting the drug distribution in MR-based human brain tumor models. These predictions showed good agreement with experimental results and this approach provides an opportunity for optimizing treatments in a patient-specific way. Recently, CFD-based software also proved useful in improving treatment planning predictions for bladder cancer patients, where fluid convection plays an important role (Schooneveldt et al., 2016). This CFD-based software developed by our group provides a useful basis for extension toward treatment planning software for HIPEC.

A first important step in the development of treatment planning software for HIPEC is to obtain insight into the influence of specific parameters. Several studies were performed on the effect of increased IFP on drug delivery dynamics, mostly focusing on intravenous drug delivery (Soltani & Chen, 2011, 2012; Zhan & Xu, 2013). These approaches focus on the drug diffusion in tissues. However, incorporating only diffusion is not sufficient for HIPEC treatments, where convection plays a crucial role in the drug distribution in the peritoneal cavity. There are very few studies using CFD-software investigating the dynamics during HIPEC treatments. Steuperaert et al. (2017) focused on the application in intraperitoneal drug delivery looking into the effect of vascular normalization for singular three-dimensional tumor models, without modeling an explicit exterior. However, convection in the peritoneal cavity affects the drug delivery, which was not accounted for during this study. Furthermore, the thermal effect during HIPEC is expected to play a major role in the drug delivery, since temperature influences the cytotoxicity of the chemotherapy, the diffusivity and the tumor microenvironment (Helderman et al., 2019, 2020). Other treatment parameters such as dose and velocity could also influence the drug distribution in the peritoneal cavity. Considering all these influences, heterogeneity of the drug distribution across the tumor surface should be accounted for. Therefore, adequate prediction of the drug delivery during HIPEC requires not only to determine the diffusion inside the tumor, but also the drug distribution at the tumor surface.

In this study we present the first three-dimensional CFD model for a single tumor nodule embedded in healthy intestinal tissue while explicitly modeling an exterior mimicking the peritoneal cavity, thus capable of addressing the shortcomings of earlier reports on this subject. We need extensive data to simulate the physical properties of chemotherapeutics. Cisplatin is one of the most investigated and experimented chemotherapies also used for HIPEC, providing the data needed to perform the simulations. The modeled exterior is used to simulate physical environments encountered in the peritoneal cavity during HIPEC with varying tumor shape and size, flow velocity, temperature and cisplatin concentration and their effect on the cisplatin distribution in tumor nodules. We determined the IC50 values of cisplatin using clonogenic assays on RKO colorectal cell lines for a range of hyperthermic temperatures and used these values to compare the numerically simulated drug distributions.

## Materials and methods

### Model geometry

**Figure 1.**
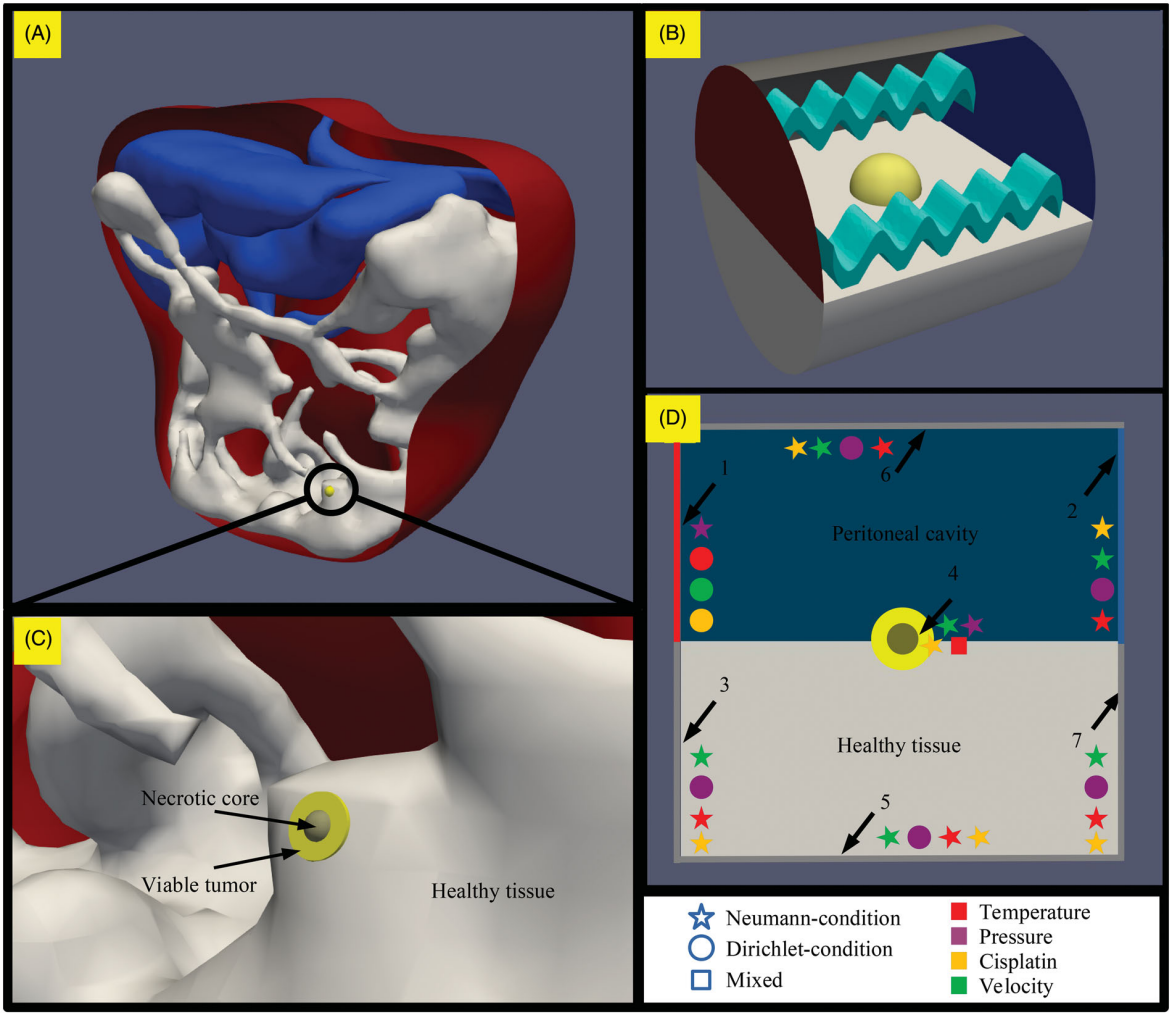
Geometry, methods and boundaries considered in this study. (A) A model representing the peritoneal cavity with a microscopic peritoneal nodule on the intestinal tract of the patient. (B) An enlarged version showing the three different types of tissue modeled in this study; healthy intestinal tissue, viable tumor tissue and a necrotic region at the center of the tumor. The tumor is embedded half way into the healthy tissue for all geometries. The size of the tumor varied from 1 to 4 mm. (C) The model during the simulation, where the yellow tumor embedded in the healthy white intestinal tissue is exposed to flow conditions relevant for HIPEC. The fluid, depicted by the waves, flows from the red side on the left to the blue side on the right. (D) The various boundary conditions encountered in the model which are explained in the boundary condition section.

During HIPEC, the limited space between the viscera is distended by the perfusate, allowing a flow to arise over the peritoneal surface. Models therefore comprised a pipe-like structure mimicking the distended peritoneal cavity allowing fluid to flow over a necrotic core encapsulated by a viable tumor nodule embedded in healthy intestinal tissue. The tissues were modeled as a porous medium. The pores represent the fluid interstitium where drug interactions take place with the solid cells. Figure 1(A) shows a model representing microscopic nodule on the large intestine left after an incomplete cytoreductive surgery. In Figure 1(B), we zoom in and illustrate the three different tissues that are featured in this study: a solid necrotic core encapsulated by a viable tumor region embedded in healthy intestinal tissue. Figure 1(C) demonstrates the simulation itself, where the perfusate, depicted by the waves, flows over the tumor nodule from the inflow depicted in red (left), to the outflow depicted in blue (right).

**Figure 2.**
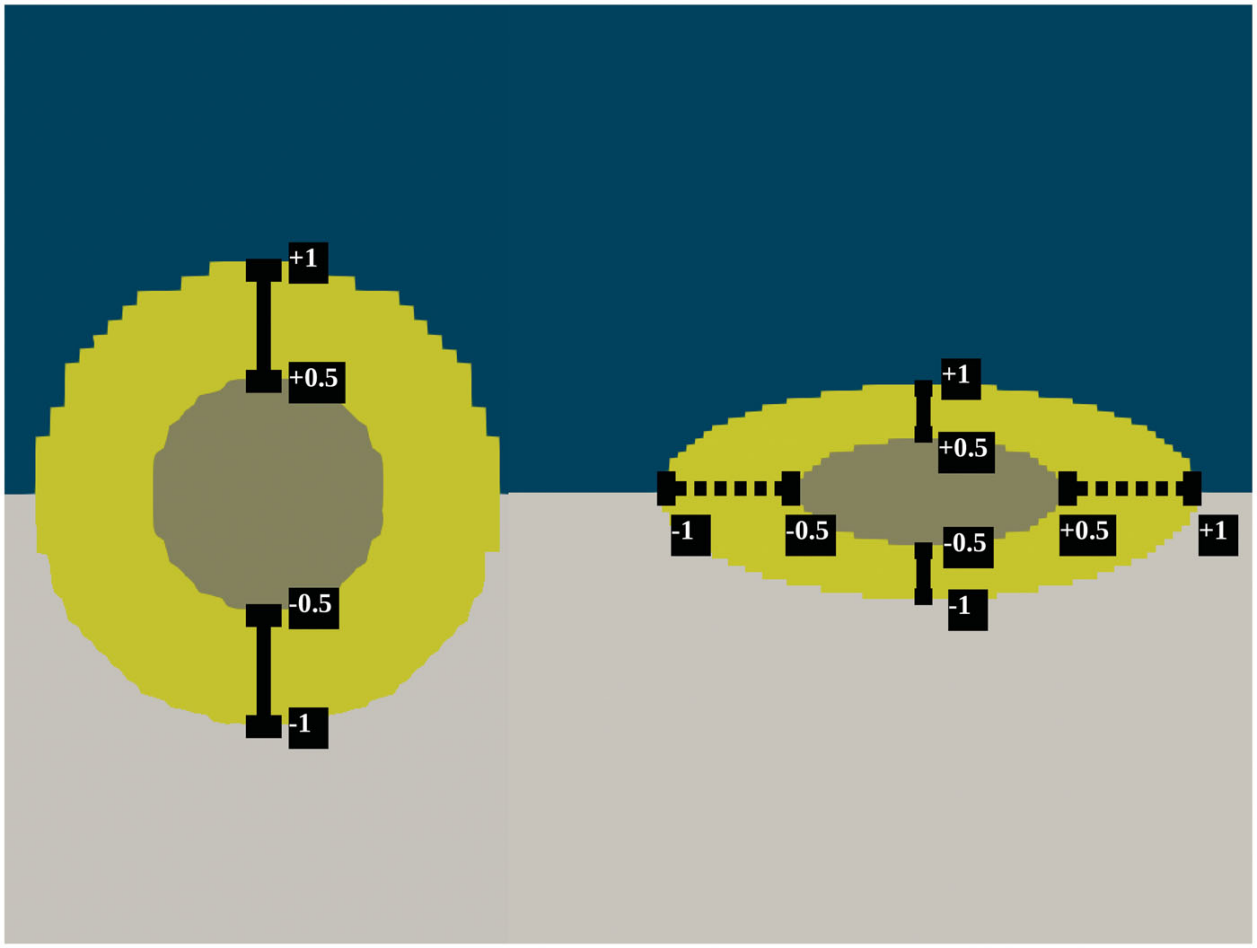
Schematic overview of the acquisition of data from the three-dimensional tumor models. The taupe colored necrotic tumor core is encapsulated by the yellow viable tumor tissue which is embedded in the healthy intestinal tissue while fluid flows over the tumors along the horizontal axis. Spherical models are probed along the vertical axes, seen on the left side while the spheroid models are probed along the vertical and horizontal axes, in this paper also referred to as minor and major axis, respectively. The coordinates represent normalized coordinates, normalized by the total length of the probe-line. These coordinates are also used in Figure 5 for an adequate comparison of the pressure and drug profiles for different tumor sizes.

Based on the scoring system of cytoreductive surgery, centering around the 2.5** **millimeter mark, we selected the sizes 1** **millimeter, 2** **millimeter and 4** **millimeter for spherical tumors. Because of the varying shapes of nodules observed in the peritoneal cavity we also considered spheroid tumor nodules, all sized 2.5** **mm along the major axis and 0.5** **millimeter, 1** **millimeter and 2** **millimeter along the minor axis. See Figure 2 for the orientation of the major and minor axes. We fixed the ratio between the necrotic core, exterior and tumor size to minimize scale-dependent effects between models. For spherical tumor nodules we used the ratio D=L=8R=16r, where *D* is the diameter of the pipe, *L* is the length of the pipe, *R* is the radius of the total nodule and *r* is the radius of the necrotic core. For spheroid nodules we used the ratios D=16R2=32r2 and L=16R1=32r1, where *D* is the diameter of the pipe, *R*2 is the radius of total nodule along the minor axis, *r*2 is the radius of the necrotic core along the minor axis, *L* is the length of the pipe, *R*1 is the radius of the total nodule along the major axis and *r*1 is the radius of the necrotic core along the major axis. Radii considered in this study are R=0.5,1,2 millimeter, R1=1.25 millimeter and R2=0.25,0.5,1 millimeter. The healthy intestinal tissue fills half of the exterior, with dimensions $\frac{1}{2}D\times L.$

### Numerical methods

We used the open source *OpenFoam* (version 6) software package for numerical modeling. Geometries as described in the previous section were designed using *SALOME-Meca* (Electricité de France, 2017) and meshed using *OpenFoam* utility *snappyHexMesh* (Weller et al., 1998). We developed dedicated solvers by augmenting the *chtMultiRegionFoam* solver with various equations to take all relevant physical processes into account, following a segregating strategy for solving equations. The solver was based on the PIMPLE algorithm, a combination of the PISO (Pressure Implicit with Splitting of Operator) and SIMPLE (Semi-Implicit Method for Pressure-Linked Equations) algorithms. The *Crank-Nicolson* scheme was chosen for the time discretization. For the finite volume discretization, responsible for interpolating the cell center values to face values, a linear *cellLimited* scheme was used, bounding the face value by the minimum and maximum neighboring cell values. The divergence terms, e.g. terms involving ∇·’s, were discretized using the Quadratic Upstream Interpolation for Convective Kinematics (QUICK) scheme, resulting in stable and accurate solutions (Leonard, 1979). Only transient simulations were performed, where a drop of four orders was considered to be a sufficient convergence criteria.

### Mathematical model

The model for cisplatin transport in the interstitium was based on the work by Baxter& Jain (1989), describing the role of convection due to an increased interstitial pressure in the motion of macro-molecules through the interstitium. We differentiated two distinct fluid dynamic regions, a free flow region in the peritoneal cavity and the fluid flow in the various tissues. The model described in this study featured three different types of tissue: healthy intestinal tissue, viable tumor tissue and a solid necrotic tumor region. The necrotic core was modeled as a solid region due to the limited fluid flow in this region and to focus on the fluid dynamics in the viable tumor region. Below, the mathematical structure is discussed per region. We describe the general equations solved during the calculations. We refer for a more detailed description of the chtMultiRegionFoam solver to (OpenFoamWiki, 2020).

### General equations

The equations governing the fluid dynamics are derived from the Navier–Stokes equation and can be described by the momentum and mass conservation equations:

(1) $$\frac{\partial }{\partial t}\rho \vec{U}=-\nabla \cdot \left(\rho \vec{U}\times \vec{U}\right)-\nabla \cdot \tau -\nabla p+\rho \vec{g},$$

(2) $$\frac{\partial \rho }{\partial t}=-\nabla \cdot \left(\rho \vec{U}\right),$$

where U,ρ,τ,p,g are the velocity [*m*/*s*], density [$kg/m^{3}$], shear-rate tensor [$kg/m/s^{2}$], pressure [*Pa*] and the gravitational vector [$m/s^{2}$], respectively. The energy equation is given by

(3) $$\frac{\partial \rho h}{\partial t}+\nabla \cdot (\rho \vec{U}h)+\frac{\partial \rho K}{\partial t}+\nabla \cdot (\rho \vec{U}K)-\frac{\partial p}{\partial t}=-\nabla \cdot \vec{q}+\rho S_{\mathrm{therm}},$$

where a heat flux is assumed, defined by $\vec{q}=-\alpha_{\mathrm{eff}}\nabla e.$ The enthalpy [$m^{2}/s^{2}$], *h* is defined as the sum of the internal energy, *e* [$m^{2}/s^{2}$] and kinematic pressure $\frac{p}{\rho }:$

(4) $$h=e+\frac{p}{\rho }.$$

The third term in Equation (3) is the time derivative of the specific kinetic energy, which is given by $K=|{\vec{U}}^{2}|/2.$ The last term in Equation (3), *S_therm_*, is the thermal sink.

The transport of cisplatin was modeled as a passive scalar, governed by

(5) $$\frac{\partial \rho C}{\partial t}+\nabla (\rho \vec{U}C)-\nabla (\rho D\nabla C)+\rho S_{c}=0,$$

where *C* is the concentration of cisplatin [$\mathrm{mol}/m^{3}$], *D* is the diffusion coefficient [$m^{2}/s$] and *S_c_* is the sink term for cisplatin [$\mathrm{mol}/m^{3}/s$]. The use of these general equations to describe dynamics in the peritoneal cavity, healthy tissue, viable tumor and necrotic core is explained in more detail below.

### Peritoneal cavity

The peritoneal cavity can be described as a free flow region where flow can be described by Equations (1) and (2). The heat dynamics are described by Equation (3), where a heat sink *S_therm_* is zero. For the transport of cisplatin in the peritoneal cavity the sink term *S_c_* in Equation (5) is also zero and we can effectively ignore the diffusion term, ∇(ρD∇C), since convective forces are dominant in the peritoneal cavity. Therefore, the drug transport in the peritoneal cavity is effectively governed by

(6) $$\frac{\partial \rho C}{\partial t}+\nabla (\rho \vec{U}C)=0.$$

### Healthy tissue

The healthy tissue region was modeled as a porous region within the peritoneal cavity. For a porous region, the momentum equation in Equation (1) can be written as

(7) $$\frac{\partial }{\partial t}\rho \vec{U}=-\nabla \cdot \left(\rho \vec{U}\times \vec{U}\right)-S_{p},$$

where the sink term *S_p_* is given by

(8) $$S_{p}=-\left(\mu D_{d}+\frac{1}{2}\rho tr(\vec{U}\cdot \vec{I})\vec{F}\right)\vec{U},$$

where *μ*, *D_d_* and *F* are the viscosity [kg/m/s], Darcy coefficient $\left[m^{-1}\right]$ and Forchheimer coefficient $\left[m^{-1}\right].$ The Forchheimer coefficient is able to account for non-linear behavior. We assumed that this non-linear behavior is negligible due to the low flow velocities inside the tissue. Therefore we write the sink term as

(9) $$S_{p}=-\left(\mu D_{d}\right)\vec{U}.$$

Note that inside the tumor, where the creeping fluid assumption is valid (i.e. advective inertial forces are small compared with viscous forces), Equation 7 simplifies into Darcy’s law: $\vec{U}=-K\nabla P_{i}.$ However, since near the boundary this assumption is not necessarily valid a general approach as in Equation (7) was used to adequately describe the dynamics inside the tumor and near the boundary.

The energy equation in the healthy tissue region is similar to Equation (3). The heat loss due to blood perfusion in living tissues was accounted for by including the blood perfusion term featured in the Pennes bio-heat equation (Valvano, 2006). Assuming that the metabolic heat rate is negligible, the sink term in Equation (3) can be written as

(10) $$S_{\mathrm{therm}}=w_{b}c_{b}\left(T_{v}-T_{i}\right),$$

where $w_{b},c_{b},T_{v},T_{i}$ are the mass flow rate of blood per unit volume of tissue [$kg/s/m^{3}$], blood specific heat [J/kg/K], blood temperature [*K*] and interstitial temperature [*K*], respectively.

Equation (5) describes the drug transport including the diffusion term ∇(ρD∇C) and the sink term *S_c_*:

(11) $$\frac{\partial \rho C}{\partial t}+\nabla (\rho \vec{U}C)+\nabla (\rho D\nabla C)+\rho S_{c}=0.$$

The sink term *S_c_* comprises all terms that can remove cisplatin from the interstitium, discussed in more detail below. The diffusion coefficient in Equation (11) depends strongly on temperature, as reflected in the approximation

(12) $$D=\frac{k_{B}T}{\gamma \eta (T)r},$$

where $k_{B},T,\eta (T),r,\gamma$ are the Boltzmann constant [*J*/*K*], the temperature [*K*], temperature dependent viscosity of the solvent [Pa·s], the radius of the spherically approximated cisplatin molecule [*m*] and a dimensionless constant, respectively. We assumed the general dependence of *D* on temperature, viscosity and radius, and fix the constant *γ* to an frequently used value assumed to be valid at T=316.15K (43 °C). For a summary of cisplatin properties used in this study, we refer to Table 1.

**Table 1.** Cisplatin diffusion properties.

	D [$m^{2}/s$] (Steuperaert et al., 2017)	D [$m^{2}/s$] Equation (11) (*γ* = 49 and T=310.15K)	D [$m^{2}/s$] Equation (11) (*γ* = 49 and T=316.15K)	r [*m*] *from* Panczyk et al. (2013)
Cisplatin	$2.5\times 10^{-10}$	$2.1\times 10^{-10}$	$2.5\times 10^{-10}$	$5.8\times 10^{-10}$

Healthy tissue also functions as a cisplatin sink, absorbing part of the cisplatin present in the peritoneal cavity, consequently reducing the cisplatin available to the tumor. The vasculature present in the tissue carries away cisplatin to the central compartment. This process is taken into account by the sink term, *S_c_*, in Equation (11), comprising al processes that can remove cisplatin from the interstitium. We can decompose the sink term, *S_c_*, into two parts: $S_{c}=S_{\mathrm{cell}}+S_{v},$ where *S_cell_* is the cellular uptake and *S_v_* is the vascular absorption. The cellular uptake can be written in terms of a first order elimination constant *β* $\left[s^{-1}\right]$ as (Steuperaert et al., 2017)

(13) $$S_{\mathrm{cell}}=\beta C,$$

representing the interaction between the interstitial and intracellular space. The vascular absorption term is more complicated and is defined as (Baxter & Jain, 1989)

(14) $$S_{v}=F_{v}\left(1-\sigma \right)C_{v}+\frac{P_{c}S}{V}\left(C_{v}-C\right)\frac{Pe_{v}}{e^{Pe_{v}}-1},$$

where $\sigma ,C_{v},P_{c}$ and *Pe_v_* are the reflection coefficient [−], concentration in the capillaries [$\mathrm{mol}/m^{3}$], permeability of the capillary wall [*m*/*s*] and the Péclet number [−], respectively. The Péclet number reflects the ratio between the convective transport and the diffusive transport across the capillary wall, and is given by

(15) $$Pe_{v}=\frac{F_{v}\left(1-\sigma \right)V}{P_{c}S}.$$

Assuming the vascular concentration to be negligible since ratios of the perfusate over the vascular drug concentration are well above 20 for a large part of the treatment (Cashin et al., 2013; Petrillo et al., 2019). We can write the total sink term as

(16) $$S_{v}=\left(\beta -\frac{P_{c}S}{V}\cdot \frac{Pe_{v}}{e^{Pe_{v}}-1}\right)C.$$

In healthy tissue, an interstitial fluid flows from the vasculature to a sink, for example the lymphatics. The total fluid accumulation inside the tissue is given as the difference between the volumetric flow leaving the capillaries, *F_v_*, and the flow entering the lymphatic system, *F_L_*. The fluid gain inside the tissue can be found by considering Starlings hypothesis stating that the volume of the fluid flow across the capillary wall is proportional to the pressure gradient over the wall, a result from the osmotic and hydraulic pressure balance between the interior and exterior of the capillary wall. As a result, the volumetric flow per unit volume $\left[s^{-1}\right]$ across the capillary wall can be written as (Baxter & Jain, 1989)

(17) $$F_{v}=\frac{L_{p}S}{V}(P_{v}-P_{i}-c(\pi_{v}-\pi_{i})),$$

where *L_p_* is the hydraulic conductivity of the vasculature [m/Pa/s], *P_v_* is the blood pressure [*Pa*], *P_i_* the interstitial pressure [*Pa*], *π_i_* is the osmotic pressure in the interstitium [*Pa*], *π_v_* is the osmotic pressure in the blood [*Pa*], *c* is a non-dimensional osmotic reflection constant and *S*/*V* is the surface area per unit volume for transport in the healthy tissue [$m^{-1}$], assumed to be uniform over the entire tissue. In healthy tissue, the lymphatic drainage is generally not zero, i.e. $F_{L}\neq 0.$ We included a term to account for the fluid drainage:

(18) $$F_{L}=-\frac{L_{p,L}S_{L}}{V_{L}}\left(P_{L}-P_{i}\right).$$

Where $L_{p,L},{\left(S/V\right)}_{L}$ and *P_L_* are the hydraulic conductivity of the lymphatics, the surface per unit volume for the lymphatics and the pressure inside the lymphatic vessel. The resulting fluid interaction was implemented as a mass/density source.

### Viable tumor tissue

The mathematical structure of the viable tumor tissue was similar to the structure used in the healthy tissue. We also modeled the viable tumor tissue as a porous medium, including similar drug and heat sinks with different physical constants (Table 2). The lymphatic system in tumor tissue is not fully functioning (Heldin et al., 2004) and we assumed that this system is entirely absent such that *F_L_* = 0, maximizing fluid accumulation. However, inside the viable tumor region $F_{v}\neq 0$ causing a fluid flow toward the exterior of the tumor according to Equation (17).

**Table 2.** Parameters used for simulations.

Parameter	Value	Reference
$L_{P}[m/Pa/s]$	$2.10\times 10^{-11}$	Baxter & Jain (1989)
$L_{P}[m/Pa/s]$	$2.70\times 10^{-12}$	Baxter & Jain (1989)
$S/V[m^{-1}]$ (Tumor)	$2.00\times 10^{4}$	Baxter & Jain (1989)
$S/V[m^{-1}]$ (Healthy tissue)	$7.00\times 10^{3}$	Baxter & Jain (1989)
$L_{P,L}{\left(S/V\right)}_{L}[m^{-1}]$	$6.91\times 10^{-8}$	Baxter and Jain (1990)
$k[m^{2}]$ (Tumor)	$2.42\times 10^{-17}$	Baxter & Jain (1989)
$k[m^{2}]$ (Healthy tissue)	$5.00\times 10^{-17}$	Baxter and Jain (1990)
$P_{v}[Pa]$	$2.08\times 10^{3}$	Baxter & Jain (1989)
$P_{L}[Pa]$	$1.33\times 10^{2}$	–
$\pi_{i}[Pa]$ (Tumor)	$2.00\times 10^{3}$	Baxter & Jain (1989)
$\pi_{i}[Pa]$ (Healthy tissue)	$1.33\times 10^{3}$	Baxter & Jain (1989)
$\pi_{v}[Pa]$	$2.67\times 10^{3}$	Baxter & Jain (1989)
*c*	0.82 (Tumor)	Baxter & Jain (1989)
*c*	0.91 (Healthy tissue)	Baxter and Jain (1990)
$\beta [s^{-1}]$	$7.32\times 10^{-4}$	Steuperaert et al. (2017)
*σ*	0.95	Baxter & Jain (1989)
$P_{c}[m/s]$ (Tumor)	$1.43\times 10^{-6}$	Baxter & Jain (1989)
$P_{c}[m/s]$ (Healthy tissue)	$1.49\times 10^{-7}$	Baxter & Jain (1989)
$c_{b}[J/kg/K]$	3500	Lang et al. (1999)
$w_{b}[ml/\min/kg]$ (Tumor)	17	–
$w_{b}[ml/\min/kg]$ (Intestine)	765	Hasgall et al. (2018)
$T_{v}[\;{}^{\circ}C]$	37	Lang et al. (1999)
$\rho [kg/m^{3}]$ (Intestine)	1088	Hasgall et al. (2018)
$p_{b}[Pa]$	0	–
$C_{b}[mM]$	0.2	–
$T_{b}[\;{}^{\circ}C]$	42	–
$v_{\max}[m/s]$	1.1	–

### Necrotic core

The necrotic core was assumed to be a solid region. Therefore, only the energy Equation (3) has to be solved. The energy equation is then given by

(19) $$\frac{\partial (\rho h)}{\partial t}=\frac{\partial }{\partial x_{j}}\left(\alpha \frac{\partial h}{\partial x_{j}}\right),$$

where *h* is the specific enthalpy, *ρ* is the density and *α* is the thermal diffusivity given by

(20) $$\alpha =\frac{\kappa }{c_{p}},$$

where *κ* and *c_p_* are the thermal conductivity and the specific heat capacity, respectively. All physical constants are given in Table 2.

### Boundary conditions

In general, the boundary conditions used in this study can be described by Neumann and Dirichlet conditions or a combination of the two. The Neumann condition can be written as

(21) $$\Gamma_{f}=\Gamma_{c}+\Delta \nabla \Gamma_{\mathrm{ref}},$$

where Γ*_f_*, Γ*_c_* and Γ*_ref_* are the face value, cell value and reference gradient of field Γ. When $\Gamma_{\mathrm{ref}}=0,$ we refer to this boundary condition as zero-gradient. The Dirichlet condition can be written as

(22) $$\Gamma_{f}=\Gamma_{\mathrm{ref}},$$

where Γ*_f_* and Γ*_ref_* is the face value and reference value, respectively. The boundary condition between solid and fluid regions was a combination of the Neumann and the Dirichlet boundary condition

(23) $$\Gamma_{s}=\Gamma_{f},\;\;\frac{\partial \Gamma_{f}}{\partial n}=\frac{\partial \Gamma_{s}}{\partial n},$$

such that the value and the gradient are equal on both sides of the interface.

We defined 7 boundary conditions in our model. Conditions were implemented for the inlet, outlet, edges and the interface between the solid necrotic core and porous viable tumor region. The tissues were implemented as porous regions within the peritoneal cavity. The high porosity, reflected by the parameter *k* in Table 2 (Baxter & Jain, 1989), ensures that the velocity is not continuous across this interface. Cisplatin and heat were able to flow freely across this interface. We visualize the boundary conditions in Figure 1(D). For edges of our computational domain, boundaries 3, 5, 6 and 7, we applied Neumann, Dirichlet and Neumann condition for the temperature, pressure and velocity fields, respectively. These boundary conditions assume no influence of the boundary on the temperature and velocity fields. The fixed value for the pressure field resulted in computational stability and is justified because we do not expect large pressure fluctuations to occur during a HIPEC treatment. Boundary 1, the inlet, was used to set the flow parameters. The inlet velocity and temperature are fixed to the treatment values according to the Dirichlet boundary condition. The Neumann condition was set for the pressure field such that the face value is equal to the adjacent cell value. The outlet value, boundary 2, was only fixed for the pressure field. The boundary conditions for the temperature and the velocity were set to Neumann conditions, projecting the local internal values to face values at the boundary. The last boundary condition applied in our model was the interface between the solid necrotic core and the viable tumor region, boundary 4. Heat should be able to flow through this interface. Therefore, we applied a combination of Neumann and Dirichlet boundary conditions to ensure that the value and the gradient are equal on both sides of the interface. The pressure and velocity boundary value were assumed equal to the local internal value according to the Neumann condition. The boundary conditions for cisplatin were of Neumann type for boundary 2–7. At the inlet, we specified the treatment concentration according to the Dirichlet boundary condition.

### Parametrical setup

The model described above allows to test the impact of various relevant parameters, thereby generating a range of possible physical environments relevant for HIPEC treatments. We tested the impact of various parameters for the largest tumor model, 4** **mm in size. Parameters introduced in the introduction are size, concentration, velocity and temperature. The first environment we considered was the baseline case, with baseline temperature, fixed concentration and fixed velocity. Baseline simulations were executed for all geometries. Baseline parameters are listed in the last four rows of Table 2. The baseline concentration was calculated using a clinical cisplatin dose of 120 $mg/m^{2}$ and a perfusate volume of 2 $L/m^{2},$ both common in clinical settings (Helderman et al., 2019). Baseline temperature was chosen to be 42** **°C (Helderman et al., 2019). The relevant flow velocity range was based on velocities known to be present during HIPEC treatments. The minimal velocity is near zero and the maximal velocity can be found directly behind the inflow catheter. Using the inner-radius of 3.1** **mm for a standard Tenckhoff catheter (Ash, 2003) and a volumetric flow rate of two liter per minute we found a maximal velocity of $v_{\max}\approx \;1.1\;m/s,$ being the inflow velocity. The different cases considered in this study are listed in Table 3.

**Table 3:** Definition of cases featured in this study. Cases are named after the parameter being tested, listed in the first column.

	Velocity [*m* / *s*]	Concentration [*mM*]	Temperature [°*C*]
Baseline	0.03	0.2	42
Velocity	0.001, 0.01, 0.1, 0.25	0.2	42
	0.75, 1.0, 2.5, 7.5, 10		
Concentration	0.03	0.2, 0.4, 0.8	42
Temperature	0.03	0.2	37, 41, 42, 43

The parameters were only tested on the largest tumor model, 4** **mm in size. The maximal clinical flow velocity was calculated to be $v_{\max}\approx 1.1\;m/s.$

### Flow profile

Convection is predominantly responsible for the distribution of heat and cisplatin in the peritoneal cavity. The actual delivery of cisplatin to the tissue is determined at the boundary layer on the surface of the tissues. A turbulent, unsteady boundary layer can occur at high flow velocities. To determine whether this is the case, we can calculate the Reynolds number

(24) $$Re=\frac{V\cdot D}{\nu },$$

where *V*, *D* and *ν* are the flow velocity [*m*/*s*], characteristic length [*m*] and the kinematic viscosity of the fluid [$m^{2}/s$]. The baseline flow velocity considered in this study was 0.03 m/s. If we take the characteristic length to be the interaction length, i.e. the diameter of the largest tumor (4 *mm*), and the viscosity of the fluid to be approximately $6.4\times 10^{-7}$ we find

(25) $$Re_{\max}=\frac{0.03\cdot 4\times 10^{-}3}{6.4\times 10^{-}7}\approx \;200.$$

A turbulent transition depends on the geometry of the flow region. For a fully developed pipe flow, the transition occurs around *Re* = 2300. Inside the boundary layer, the transition occurs between $3.5\times 10^{5}$ and 10^6^ for flow over a flat plate. This critical values decreases to $3\times 10^{5}$ for flows over blunt bodies, such as cylinders and spheres (Schlichting, 1979). Our geometry can be considered as a combination of these three geometries. However, our baseline Reynolds number does not exceed any of the critical Reynolds numbers mentioned. Therefore, we can justify the assumption of laminar flow. For velocity cases featuring flow velocities up to 10 *m*/*s* the Reynolds number is around 300 times larger ($Re\approx 6\times 10^{4}$). We assume that the boundary layer is still laminar and we evaluated the cisplatin distribution on the surface of the tumor.

### Grid independence and validation

A mesh sensitivity analysis was performed to ensure the grid size independence of our results. Computational grids included hexahedral and polyhedral elements. Grids used for the tumor region consisted out of 50900,120120 and 735000 cells for the low, medium and highly refined meshes, respectively. We compared intratumoral parameters such as the pressure gradient and the velocity distribution.

The intratumoral pressure distribution, simulated in the 4 mm tumor nodule, was validated using values found in Boucher et al. (1990).

### Determination of temperature-dependent IC50 values for cisplatin

In this study, we calculated the effective penetration of cisplatin in tumor nodules using the IC50 values for cisplatin measured on RKO colorectal cell lines at the temperature relevant for the specific case. These values were used in our computational models to calculate the effective penetration depth. The IC50 value is defined as the drug concentration needed to reduce cell survival by 50%. The effect of the enhanced cytotoxicity of cisplatin due to heat was evaluated using clonogenic survival assays. More specifically, RKO cells were cultured in McCoy’s 5 A medium supplemented with 25 micromolar of 4-(2-hydroxyethyl)-1-piperazineethanesulfonic (HEPES) acid, 10% fetal bovine serum and 1% penicillin/streptomycin/glutamine. Cells were maintained at 37 °C in a humidified atmosphere of 5% *CO*_2_ in air. Clonogenic survival assays were performed to find the thermal enhancement of cisplatin at three different elevated temperatures, compared to 37 °C. Cells were harvested, counted and plated in appropriate densities in 6-well plates (Greiner). We used 250, 500, 1000 and 1000 cells for the different treatment groups: 37 °C, 41 °C, 42 °C and 43 °C, respectively. Plates were incubated at 37 °C. The next day, HIPEC was mimicked in an *in vitro* setting by exposing the RKO cells to 0, 2, 4, 8, 12, 16, 18 and 20 micromolar of freshly made stock solution cisplatin (Platosin, Pharmachemie) under hyperthermic conditions for 60 minutes. Hyperthermia was performed by placing the plates in a thermostatically regulated water bath (Lauda aquiline AL12, Beun de Ronde, Abcoude, The Netherlands) supplemented with *CO*_2_. Immediately after treatment, medium containing chemotherapy was replaced with complete fresh medium and the cells were incubated for 10 days for colony formation at 37 °C in a humidified atmosphere of 5% *CO*_2_ in air. After 10 days, colonies were fixed by 6% glutaraldehyde and stained with 0.05% crystal violet. Colonies of 50 cells or more were scored as originating from a single clonogenic cell (Franken et al., 2006). The surviving fractions were calculated and corrected for plating efficiency. Surviving fractions calculated from 4 independent experiments were used to create graphs of the cell survival as function of the cisplatin concentration for each temperature level and the IC50 values and thermal enhancement ratios were determined. The thermal enhancement of cisplatin is reflected by the thermal enhancement ratio (TER), defined as the ratio of cisplatin dose required to reach a specific end point at 37 °C over the dose of cisplatin required to reach the same end point at an enhanced temperature level (Overgaard, 1984). Our end point was chosen to be at 50% cell-survival.

## Results

We first present the results from the clonogenic assays and the grid independence study. We then discuss the results from the simulation study per case as defined in Table 3. Calculated cisplatin distributions of our computational models were compared by evaluation of the effective penetration depth, as determined by the IC50 value. To this end, the experimentally determined IC50 values of cisplatin were used as reference values.

### IC50 values and TER-values

The *in vitro* experiments showed that thermal enhancement ratio for cisplatin increases with temperature for the considered temperature range (37 °C, 41–43 °C) resulting in decreased IC50 values for higher temperatures. The TER and IC50 values, as determined from clonogenic assays, are listed in Table 4.

**Table 4.** IC50 values and thermal enhancement ratio (TER) of cisplatin for RKO cells, determined from clonogenic assays.

Temperature	37 °C	41 °C	42 °C	43 °C
IC50 [*μ*M]	19.5	11.2	9.9	6.8
TER	1.0	1.7	2.0	2.9

### Grid independence & validation

**Figure 3.**
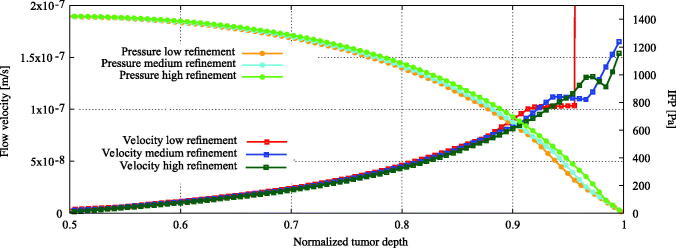
Mesh sensitivity analysis. Computational grids include hexahedral and polyhedral elements. Grids used for the tumor region consisted out of 50900,120120 and 735000 cells for the low, medium and highly refined meshes. The study focused on the relevant intratumoral parameters such as the interstitial pressure and velocity distribution. The distributions for interstitial fluid pressure (IFP) and velocity were similar for all three grid resolutions. Only the low refined mesh was unable to correctly simulate the velocity gradient near the edge of the tumor, illustrated by the vertical red line.

The distributions for interstitial pressure and velocity were largely similar for all three grid sizes (Figure 3). Only the coarse mesh was unable to compute the velocity gradient near the edge correctly. The results in this study can thus be regarded grid independent and the medium refined mesh should be regarded as to give sufficiently accurate results. Therefore, the medium refined mesh was used in the remainder of this study.

**Figure 4.**
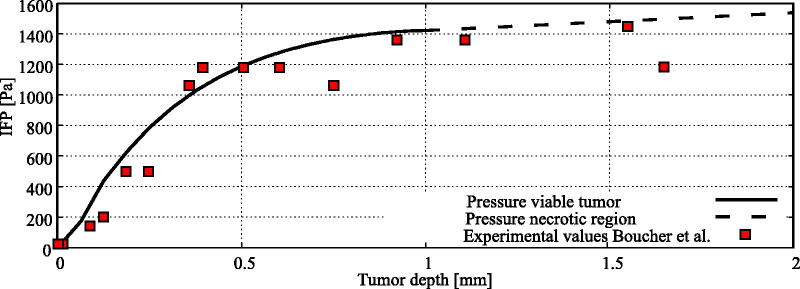
Experimental comparison for the interstitial fluid pressure (IFP) profile in the 4 millimeter tumor nodule considered in this study with experimental data from Boucher et al. (1990). The interstitial pressure profile inside the necrotic region was linearly interpolated between boundaries of the necrotic and viable tumor tissue.

The interstitial pressure profile of the 4 millimeter model compared very well to the experimentally determined interstitial pressure profile from (Boucher et al., 1990) (Figure 4).

### Baseline

**Figure 5.**
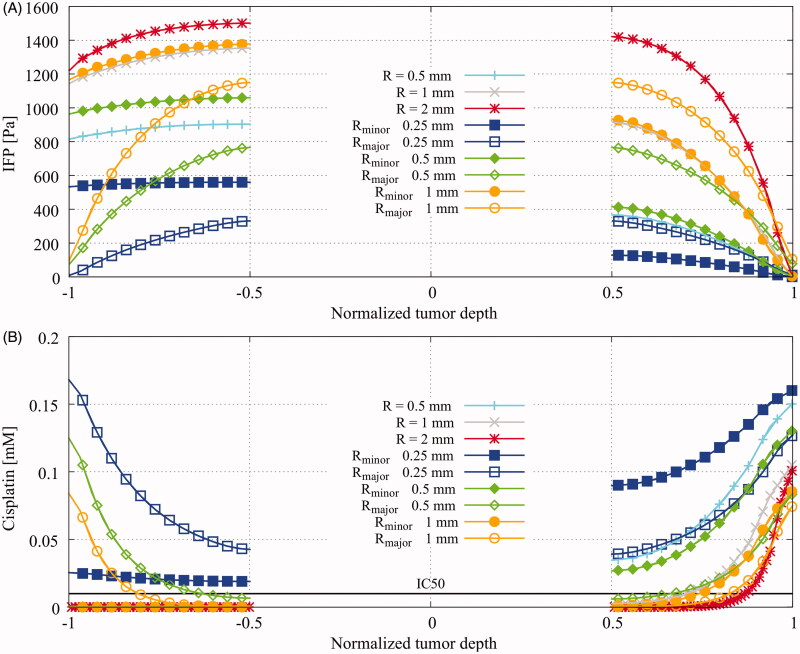
Baseline results showing (A), interstitial fluid pressure (IFP) profiles in *Pa* versus the normalized penetration depth and (B), cisplatin distributions in *mM* versus the normalized penetration depth. Spherical radii considered were 0.5, 1 and 2 millimeter. Spheroid tumor sizes considered were 0.25, 0.5 and 1 millimeter along the minor axis (MiA) with a fixed radius along the major axis (MaA) at 1.25 millimeter. The tumor depth on the horizontal axis was normalized using the total length of the probe line, depicted in Figure 2. For spherical and spheroid tumors along the minor axis, the normalized tumor depth −1 can be interpreted as the edge between the viable tumor region and the normal tissue region and normalized tumor depth +1 can be interpreted as the boundary between viable tumor tissue and the peritoneal cavity. The major axis was oriented along the flow direction, orthogonal to the vertical axis.

Figure 2 shows how the data, presented in the remainder of this section, were extracted from the simulation. Probing is always done along the vertical axes of the spherical tumor. The spheroid tumor nodule is also probed along the horizontal axis, parallel to the flow direction. The probe lines run from the exterior of the tumor toward the edge of the necrotic core and from the necrotic core to the boundary between tumor and healthy tissue. The baseline simulation was performed for all geometries, showing the effect of size and shape on the relative effective penetration depth and interstitial pressure distribution. The interstitial pressure profiles and concentration distributions along the probe lines are shown in Figure 5(A) and 5(B), respectively. The relative depth at which the IC50 of cisplatin value was achieved increased when the size of the viable tumor region decreased. Shape of the nodule was also a relevant factor, where for example the 2 millimeter spheroid tumor nodule had a smaller relative penetration depth than the equally sized 2 millimeter spherical tumor. The drug accumulation inside the tumors provides a more complete picture (Table 5), showing that larger tumors correspond to higher maximal interstitial pressures and lower amounts of accumulated cisplatin. Only the smallest spheroid tumor completely received a cisplatin concentration exceeding the IC50 value.

**Table 5.** Maximal achieved interstitial pressures and average accumulated drugs in the various baseline geometries.

	Spherical	Spheroid	Spherical	Spheroid	Spherical	Spheroid
	(R** **=** **2mm)	(R** **=** **1mm)	(R** **=** **1mm)	(R** **=** **0.5mm)	(R** **=** **0.5mm)	(R** **=** **0.25mm)
Maximal interstitial pressure [Pa]	1533	1378	1357	1060	914	660
Average accumulated drugs [*μM*]	12.05	25.14	29.50	44.36	54.67	80.08

The interstitial pressure profiles of the spherical tumors show that the tumor-half adjacent to the peritoneal cavity had a higher pressure gradient than the tumor-half adjacent to the healthy intestinal tissue. In our model, the fluid drainage in the healthy tissue is fully depending on the lymphatic vessels, which are not able to instantaneously remove the fluid from the system resulting in an additional pressure build-up in this region. Maximal interstitial pressures increased for larger tumors for both the spherical and spheroid tumor nodules.

### Flow velocity

**Figure 6.**
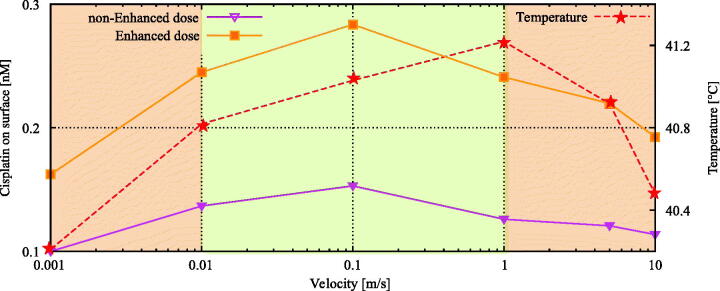
Flow velocity influence on cisplatin and thermal distributions at the tumor surface. Lower flow velocities results in a lower amount of cisplatin on the surface while higher flow velocities lower the coverage around the tumor surface. The similar phenomenon is visible for the average temperature inside the tumor. This general behavior suggest that an intermediate velocity is optimal to heat the peritoneal surface fast while maintaining sufficient cisplatin coverage. The orange line depicts the thermally enhanced equivalent dose. Based on the enhanced equivalent dose we can determine the optimal velocity range, shown by the green area, where both temperature and cisplatin coverage are high. The optimal range was determined as the range where the maximal enhanced dose is achieved.

In Figure 6 we plot the average cisplatin concentration at the tumor surface and the average achieved temperature in the 4 millimeter tumor nodule. We observed a pattern where relatively low and relatively high flow velocities resulted in reduced cisplatin concentrations at the tumor surface. Lower flow velocities deliver less cisplatin to the surface, while higher flow velocities resulted in flow patterns where the drug distribution over the tumor surface was non-uniform. The delivery is optimal in an intermediate flow velocity range.

Increased flow velocities have an impact on the heating rate of the peritoneal surface as well. Fast heating of the peritoneal surface can also limit thermal damage in deeper parts of the peritoneal organs (Furman et al., 2014). The effect of rapid heating for high flow velocities was also visible in our models. Simulations performed with low flow velocities took more time to equilibriate and the average viable tumor temperature was observed to be lower. Very high flow velocities reduced the uniform heating of the nodule, resulting in lower average temperatures.

Both processes are comparable and have different optimal velocity ranges. We can combine both curves by defining an effective dose. We can include the enhanced cytotoxicity of cisplatin, as a result of the elevated temperatures, by multiplying the concentration of cisplatin with the TER. Determining the optimal velocity range for this effective dose results in the optimal treatment velocity range, visible in Figure 6. The optimal range was determined as the range where the maximal enhanced dose is achieved. We can see from Figure 6 that the maximum occurs between 0.01 *m*/*s* and 1 *m*/*s*.

### Dose and thermal effects

**Figure 7.**
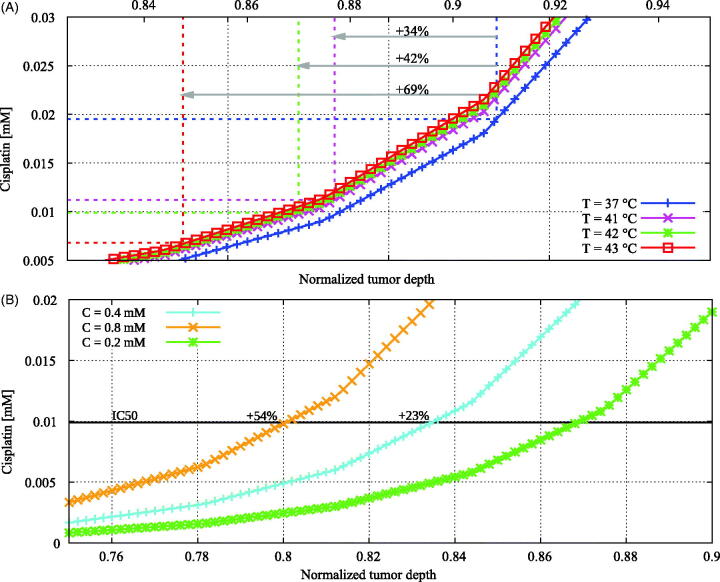
Cisplatin distributions showing the qualitative behavior of the influence of (A) temperature and (B) administered dose on the penetration depth of cisplatin. Coordinates run toward the tumor-peritoneal cavity boundary at +1. See Figure 2 for a detailed explanation of the coordinates. Using higher temperatures, a lower dose of cisplatin is needed to induce the same amount of cell death illustrated by a lower IC50 value, plotted as the dashed lines in (A). In combination with a higher diffusivity, this results in a larger penetration for higher temperatures. Higher doses result in a larger penetration depth, as is shown in (B).

Increasing the cisplatin dose in the carrier solution had the expected effect of increasing the cisplatin dose in the tumor. Comparing baseline (C=0.2 mM) with C=0.4 mM and C=0.8 mM, we observed increases in effective absolute penetration depth of 23% and 54%, respectively. See Figure 7(B) for the drug distribution along the probe line.

**Figure 8.**
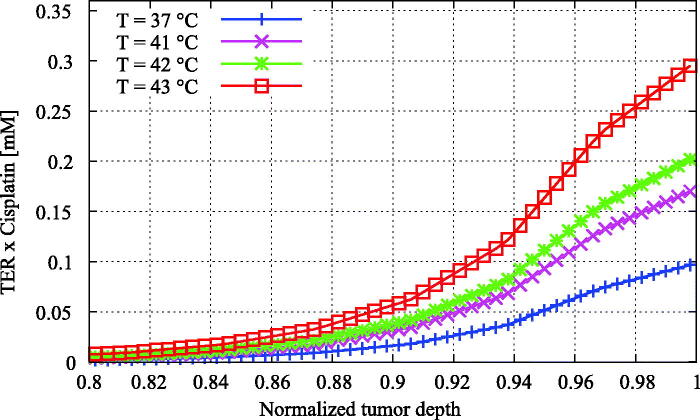
Enhanced cisplatin distributions versus the normalized tumor depth for varying temperature. The enhanced cisplatin dose was calculated by multiplying the cisplatin concentration by the thermal enhancement ratio of the temperature level (Table 4). The enhancement of the treatment effectiveness due to elevated temperatures is now more apparent than in Figure 7(A), showing higher effective doses for higher temperatures.

In Figure 7(A), we present cisplatin distributions for 37 °C, 41 °C, 42 °C and 43 °C. Diffusion constants, as calculated with Equation (12), increased with increasing temperatures, leading to effectively different drug distribution curves in Figure 7(A). We compared cisplatin distributions at the IC50 value. Since the cytotoxicity of cisplatin is thermally enhanced, IC50 values decreased for higher temperatures. Therefore, we compared cisplatin distributions at the IC50 value determined at the relevant temperature, resulting in an increased effective penetration depth for higher temperatures. Specifically, compared to normothermic temperatures, e.g. 37 °C, penetration depths were 34%, 42% and 69% higher for 41 °C, 42 °C and 43 °C, respectively. This can be interpreted as if the modeled tumor nodules reached an equivalent dose at a larger depth for higher temperatures. This phenomenon is visualized in Figure 8, where we show the thermally enhanced cisplatin dose for all four temperatures showing the added effect of temperature.

## Discussion

In this study we presented a three-dimensional tumor nodule model with a dynamic physical environment mimicking the peritoneal cavity during HIPEC treatments. Using this novel model we investigated the impact of relevant parameters on drug penetration into the tumor models. Smaller tumors were found to have lower IFP and higher cisplatin accumulation. Furthermore, we found an intermediate flow velocity range where cisplatin delivery to the surface is optimal. Also, increased concentrations and higher temperatures resulted in increased cisplatin concentrations in our 4 millimeter spherical tumor nodule model.

This study focused on the influence of tumor size and shape, drug concentrations, temperature and flow velocity on the effective penetration depth in the viable tumor tissue. We only evaluated the drug distribution in the viable tumor region and not in the necrotic core, which was assumed to be solid, i.e. without drug penetration. Although in real-life situations, cisplatin is also to some extent able to diffuse into the necrotic core, this was not taken into account in this study since previous research showed that the penetration depth is independent of the presence of an explicitly modeled necrotic core (Steuperaert et al., 2017). Therefore, we fixed the radius of the necrotic core and only solved the energy equation inside the necrotic core, which also reduced computation times. However, when evaluating other aspects, such as the pressure profile inside the tumor nodule, the exact geometry of the necrotic core will be more relevant. Soltani & Chen (2011) showed that the maximally achieved interstitial pressure increases with a decreasing radius of the necrotic core. Furthermore, the necrotic region could be heterogeneous which could alter the dynamics inside the tumor. A recent computational study investigated pressure distributions in tumor nodule models, based on MRI delineations (Steuperaert et al., 2019). The interstitial pressure profiles differed from the interstitial pressure profiles found in the simplified geometries used in this study. As a result a different dynamic is expected inside the tumor, possibly affecting drug delivery. These geometries should be incorporated in future treatment planning applications to evaluate their precise effect. The interstitial fluid pressure is also sensitive to the presence of healthy tissue, incorporated in our models. Fluid near the boundary between the tumor and the peritoneal exterior is able to flow away freely while the fluid near the boundary between the tumor and healthy tissue is not. The lymphatics cannot instantaneously remove the fluid from the healthy tissue. Therefore, an additional fluid build-up was visible near the healthy tissue boundary, visible by the asymmetry in Figure 5(A).

Maximal interstitial pressure grew for larger tumors, resulting in a lower accumulation of cisplatin. Only the smallest geometries considered, around 1 mm in size, were predicted to receive a cisplatin dose exceeding the IC50 value. This illustrates the limitations of HIPEC in treating large nodules and therefore the importance of reaching a complete cytoreduction before HIPEC is administered. Although size and shape of the tumor nodules largely determine the drug penetration, our study revealed that the effective penetration depth can be increased by optimization of different treatment parameters, such as velocity, dose and temperature.

High flow velocities resulted in flow-separated regions across the downstream half of the exposed tumor surface. The flow is nearly stationary in these regions and cisplatin concentrations are significantly lower than near the upstream tumor surface. This results, on average, in a lower cisplatin concentration over the tumor surface. In general, higher flow velocities correspond to higher Reynolds numbers, resulting in larger flow-separated regions. The onset of flow-separated regions also varies depending on Reynolds number. Flow-separated regions tend to occur increasingly upstream for higher Reynolds numbers, exemplified by Islam et al. (2014) who evaluated flow over a circular cylinder at low Reynolds numbers. Using flow velocities below the optimal range resulted in slow cisplatin delivery to the tumor surface. In that case, the tumor can process more cisplatin than the flow is able to provide near the surface. Therefore, an optimal flow velocity range can be formulated, optimizing the delivery of cisplatin to the peritoneal surface. Similar behavior was observed for the temperature distribution inside the nodule.

High flow velocities can also accelerate the heating of the peritoneal surface and consequently, of the peritoneal tumor load. Rapid heating of the peritoneal contents could protect the viscera from (deep) thermal damage (Furman et al., 2014). Our models showed that higher flow rates initially increased the heating rate in the tumor nodule, demonstrated in Figure 6. If low flow velocities are applied, the vasculature is able to carry away more heat than is supplied by the flow in the peritoneum. High flow velocities will result in flow separated regions, prohibiting a uniform temperature distribution over the tumor surface. Similarly to the delivery of cisplatin, we can determine an ideal flow velocity range where thermal distribution is optimal. The optimal velocity ranges for the delivery of heat and drugs are not necessarily the same. Therefore it is interesting to optimize the effective dose, defined by multiplying the concentration of cisplatin with the TER. Then the optimal flow velocity range can be identified as the range where the effective dose is at a maximum. In this study, the ideal flow velocity range is depicted in Figure 6 by the green shaded area. In clinical settings, the general flow velocity varies between 0.5 and 2 *L*/*min* (Helderman et al., 2019), corresponding to 1.5–6 *m*/*s* at the inflow. In flow detached regions, the flow velocity is significantly lower. It is difficult to locally determine whether the optimal flow velocity is achieved. Modeling flow patterns in the entire peritoneal cavity can help to identify regions where flow velocities are below or above the optimal range.

This behavior can be directly translated to the entire peritoneal cavity. Flow detached regions increase when flow velocities are above the optimal range. Below the optimal range, the rate at which heat and cisplatin are provided to the peritoneal surface could be insufficient. Manual agitation or manual stirring of the abdominal contents, as applied during clinical HIPEC treatments, can reduce this problem. It is difficult to incorporate the influence of the manipulation in models like the one developed in this study. However, manual agitation can be regarded as a reset of the established flow pattern. After manual agitation, another flow pattern will emerge after a certain amount of time. To reduce the dependence on the manual interventions, the optimal flow range should be determined before treatment is started. Complete modeling of the peritoneal cavity should be able to shed light on how fast these flow patterns are realized and whether changing the catheter setup can change the preferential direction of the flow pattern. It is possible that the complicated geometry of the peritoneal cavity results in a more turbulent flow. The level of turbulence expected inside the peritoneal cavity should therefore be investigated.

Increasing the dose increased the penetration depth and our results showed that doubling the dose increased the effective penetration depth by 23–54%. The cisplatin dose in our model was implemented as a fixed concentration, defined in units of moles per cubic meter. Another frequently used approach is basing the dose on the body surface area of the patient. Recent *in vivo* experiments demonstrated that the concentration in peritoneal tissues and plasma are significantly different whether a fixed concentration or a body surface area-based dose is used (Lemoine et al., 2019). A fixed concentration will result in more predictable exposure to the peritoneal structures. A body surface area-based approach will result in more easily controllable systemic toxicity, especially when varying perfusate volumes are used (Lemoine et al., 2017). This perfusate volume varies widely between different institutes, with volumes as low and high as 2 L and 12 L, respectively (Helderman et al., 2019). This could increase or decrease the treatment concentration 2- to 6-fold. To realize a uniform total dose and treatment concentration for all patients, the applied volume and dose could be based on the body surface of the patient, fixing both the total dose and the treatment concentration.

Higher temperatures resulted in higher effective penetration depths, up to an increase of 69% compared to treatments performed at 37 °C. The enhanced effective penetration for higher temperatures is a result of two different processes: higher diffusivity of cisplatin and the thermally enhanced cytotoxicity of cisplatin. The difference between the blue (+), magenta (×), green (*) and red (□) cisplatin concentration curves for 37 °C, 41 °C, 42 °C and 43 °C in Figure 7(A) reflect the influence of higher diffusivity. This effect is limited compared to the thermal enhancement of cisplatin. We compared simulations performed at different temperatures with the corresponding IC50 values (Table 4), visualized by the dotted lines in Figure 7(A). The combined effect of the enhanced diffusivity and the enhanced cytotoxicity can be visualized in a more prominent way if we consider the effective cisplatin dose. This approach is comparable to calculating the biological effective dose (BED) in bi- and trimodality treatments involving hyperthermia, radiotherapy and/or chemotherapy (Plataniotis & Dale, 2009). A straightforward way to implement this is by multiplying the cisplatin dose with the thermal enhancement ratio. Figure 8 shows the normalized tumor depth versus the effective dose of cisplatin reflecting the total enhancement of involving hyperthermia during the perfusion, which accentuates the potential additive thermal effect of the HIPEC treatment.

In case of a complete cytoreduction after surgery, remaining tumor nodules are typically below 1 millimeter in size and optimizing the flow parameters would probably be sufficient to achieve adequate penetration of cisplatin. However, when cytoreductive surgery is not complete, scoring CCR-1 or CCR-2, the penetration of cisplatin might be insufficient. Application of external intra-abdominal pressure during HIPEC is considered a viable option to increase the penetration depth. Various *in vivo* studies in rat and pig models investigated the influence of the application of intra-abdominal pressure on chemotherapy concentrations in tissue after HIPEC. It was found that drug concentrations in the peritoneal tissues could double when HIPEC was combined with intra-abdominal pressures up to 40 mmHg, more than 5000 Pa (Esquis et al., 2006; Facy et al., 2012). Adverse effects were limited when choosing appropriate drug concentrations. Recently, a pilot study was published showing that pressurized HIPEC can also be administered to patients (Sánchez-García et al., 2016). First results are promising and demonstrate feasibility, but more research is required to ensure a safe and optimal clinical application of pressurized HIPEC. Important aspects to be investigated are the maximum amount of pressure that can be safely applied and the influence on the systemic toxicity.

The diffusion constant was determined using Equation (12) and calibrated on a frequently used value (Steuperaert et al., 2017). We also assumed a direct dependence on temperature and radius of the, spherically assumed, cisplatin molecules following the Stokes–Einstein relation. The use of Equation (12) is supported by a simulation study (Panczyk et al., 2013). Approximately equal slopes were found for the Stokes–Einstein relation and the simulated values at 297 K (23.85 °C), 300 K (26.85 °C) and 310 K (33.85 °C). However, an exact determination of the diffusion constant values at different temperatures in various tissues (e.g. tumorous and healthy) can increase the accuracy of the model presented in this study. In this study a constant fluid density was assumed, thereby not accounting for gravitational effects. These gravitational effects become substantial in case of density fluctuations, which are almost absent in the peritoneum due to mixing in the peritoneal cavity. However, inside large tumors, where also temperature gradients exist, density fluctuations and thus gravitational effects might be non-negligible. Investigation of these gravitational effects is subject of further research.

The tumor model presented in this study assumed a homogeneous tumor consisting of only tumor cells, with the consequence that not all biological processes are properly taken into account. Part of the cisplatin is intercepted and deactivated before it can reach the DNA in the cell nucleus. Cisplatin is known to bind easily to intracellular- and extracellular protein and protein present in plasma is known to form stable complexes with primarily N- and S-donor rich compounds (Appleton, 1997; Messori & Merlino, 2016). The direct consequence of this protein binding is the decreased availability of free cisplatin. The remaining cisplatin is able to interact with the cancer cells. Up to 75% of the cisplatin can be bound (Bhandari et al., 2019), which could impact the absolute penetration depth. However, in this study we focused on the qualitative behavior under variations in treatment specific parameters. Incorporating the drug binding would influence all cases in a similar way and would therefore not influence the qualitative results presented in this study. For future studies, determining the penetration depth in a quantitative manner, drug binding becomes more relevant.

The interaction between the tumor cells and the interstitium was implemented by modeling the cellular uptake as a sink term in Equation (13). This is a simplification, not accounting for more complex interactions between cells and cisplatin, e.g. the back-flow of cisplatin, possibly affecting the effective penetration depth. In future studies, the complete interaction between the interstitial space and intracellular space, including protein binding, should be incorporated.

During the *in vitro* experiments, a homogeneous colony of cancer cells was treated with cisplatin to determine the IC50 value. *In vivo* conditions are known to be able to influence the IC50 value (Zeitlinger et al., 2011). The determination of the IC50 values during *in vivo* experiments could increase the clinical relevance of the results. Cisplatin is one of the chemotherapeutic agents most sensitive to thermal enhancement. This is due to the direct damage cisplatin causes on the DNA of cancer cells, and the inhibition of DNA repair induced by hyperthermia (Oei et al., 2020). Thermal enhancement ratios for cisplatin for RKO cell lines found in this study are comparable to values found in (Bergs et al., 2007), where cisplatin was applied to SiHa cell lines. Other chemotherapeutic agents, such as mitomycin-C or 5-FU, are also applied during clinical HIPEC and have different enhancement ratios (Urano & Ling, 2002). The different thermal enhancement ratio curves result in an enhanced or reduced thermal benefit, as was demonstrated in Figure 7(A) for cisplatin. Extensive *in vitro* and *in vivo* experiments, varying chemotherapy and cell-line, are needed to investigate the entire spectrum of thermal enhancement for each cancer origin. Substantial *in vitro* data, combining various chemotherapies with various cell-lines, is presented in (Helderman et al., 2020), underlining the variability of the thermal enhancement of chemotherapies.

Local and systemic toxicity are a relevant risk during the application of HIPEC. In this study, the systemic contribution to the drug delivery was considered negligible. This assumption is valid to represent the first phase of HIPEC, when systemic concentrations are still close to zero. The maximal systemic concentration of cisplatin is achieved at the end of HIPEC, but amounting to significantly lower values than the cisplatin concentration found in the perfusate. The ratio of cisplatin concentration in the perfusate over the concentration in the plasma is between 40 and 60 during the first ten minutes, declining to 8–20 at the end of the treatment (Cashin et al., 2013; Petrillo et al., 2019). Therefore, the effect of including this mode of delivery is probably small, but in future studies it would be interesting to simulate the systemic concentration as well. This could be done by modeling the complete peritoneal cavity combining local contributions to the systemic concentration, including the potential effect of temperature on the systemic toxicity. This provides insight into the possible occurrence of systemic toxicity, which can help to develop treatment strategies to limit toxicity in patients. Furthermore, one would expect that high effective doses, combining high cisplatin with high temperature, are responsible for an increased systemic toxicity. However, in a study performed by *Piché et al.*, rats were observed to have lower systemic concentrations of oxaliplatin if they were treated at higher temperatures (Piché et al., 2011), probably directly translatable to cisplatin-based treatments. This principle is one of the major advantages of HIPEC and it is important to incorporate this effect when modeling the entire peritoneal cavity.

The software presented in this study can be used as a basis for patient-specific treatment planning software. Dose, flow velocity and temperature could fluctuate over the peritoneal surface during HIPEC treatments. Therefore, each case presented in this study mimics a local possible scenario during a HIPEC treatment. This underlines the importance of the development of treatment planning software for HIPEC, able to provide three-dimensional distributions of flow patterns, temperature and chemotherapy. The location of the microscopic residual tumors is unknown. Homogeneous flow velocity, temperature and chemotherapy distributions are therefore essential for eradicating all microscopic disease. Modeling the peritoneal cavity, including extensive thermal and drug dynamic modules, will provide a tool to detect and remove possible heterogeneities. Addition of catheters, adjusting catheter positions or applying flow inversions are tools to enhance the homogeneity in the peritoneal cavity, and can be tested using treatment planning software. The effect on the systemic values, such as core temperature and systemic concentration of the chemotherapy, could also be monitored during perfusion, providing a guideline for overheating or systemic toxicity. The software can incorporate patient-specific data, such as tumor pathology, peritoneal cavity geometry after resections, body-surface and peritoneal volume, which can be determined before surgery and can help improve the accuracy for individual patients. Treatment planning software is currently under development in our group, aiming to optimize HIPEC treatments and further improve clinical results.

## Conclusion

In summary, in this study we presented the first three-dimensional CFD model for a single tumor nodule embedded in healthy intestinal tissue while explicitly modeling an exterior mimicking the peritoneal cavity. Relevant parameters were investigated which could impact the penetration depth of cisplatin into tumor nodules such as dose, temperature and velocity. Our results show qualitatively that higher temperatures, moderate flow rates and higher dose could all increase the penetration of cisplatin. Our study also underlines that HIPEC is most effective when cytoreductive surgery is complete and only nodules smaller than 1 millimeter remain present on the peritoneal surface. The results presented in this study showed that the thermal and velocity distributions are the most important factors to control the drug delivery. The influence of flow velocity suggests the need for including the entire peritoneal surface into our models to assess the distribution throughout the peritoneal cavity. These models should be able to correctly predict flow patterns and simulating flow-detached regions. We plan to extend the software presented in this study toward patient-specific treatment planning software suitable for use in HIPEC.
